# High Gastrointestinal Colonization Rate with Extended-Spectrum β-Lactamase-Producing *Enterobacteriaceae* in Hospitalized Patients: Emergence of Carbapenemase-Producing K. *pneumoniae* in Ethiopia

**DOI:** 10.1371/journal.pone.0161685

**Published:** 2016-08-30

**Authors:** Kassu Desta, Yimtubezinash Woldeamanuel, Aklilu Azazh, Halima Mohammod, Dawit Desalegn, Damte Shimelis, Dereje Gulilat, Biruk Lamisso, Eyasu Makonnen, Alemayehu Worku, Kerstin Mannerqvist, Johan Struwe, Olov Aspevall, Eleni Aklillu

**Affiliations:** 1 Department of Microbiology, Immunology and Parasitology, School of Medicine, College of Health Sciences, Addis Ababa University, Addis Ababa, Ethiopia; 2 Department of Internal Medicine, School of Medicine, College of Health Sciences, Addis Ababa University, Addis Ababa, Ethiopia; 3 Department of Gynecology & Obstetrics, School of Medicine, College of Health Sciences, Addis Ababa University, Addis Ababa, Ethiopia; 4 Department of pediatrics, School of Medicine, College of Health Sciences, Addis Ababa University, Addis Ababa, Ethiopia; 5 Department of Surgery, School of Medicine, College of Health Sciences, Addis Ababa University, Addis Ababa, Ethiopia; 6 Department of Orthopedics School of Medicine, College of Health Sciences, Addis Ababa University, Addis Ababa, Ethiopia; 7 Department of Pharmacology, School of Medicine, College of Health Sciences, Addis Ababa University, Addis Ababa, Ethiopia; 8 School of Public Health College of Health Sciences, Addis Ababa University, Addis Ababa, Ethiopia; 9 The Public Health Agency of Sweden, Stockholm, Sweden; 10 Division of clinical Pharmacology, Department of Laboratory Medicine, Karolinska Institutet, SE 141 86, Stockholm, Sweden; Ross University School of Veterinary Medicine, SAINT KITTS AND NEVIS

## Abstract

We investigated the gastrointestinal colonization rate and antibiotic resistance patterns of Extended-Spectrum Beta-Lactamase (ESBL)- producing *Escherichia coli* and *Klebsiella pneumoniae* in hospitalized patients admitted at Ethiopia’s largest tertiary hospital. Fecal samples/swabs from 267 patients were cultured on chrome agar. ESBL. Bacterial species identification, verification of ESBL production and antibiotic susceptibility testing were done using Vitek 2 system (bioMérieux, France). Phenotype characterization of ESBL-*E*.*coli* and ESBL- *K*.*pneumoniae* was done using Neo-Sensitabs™. ESBL positivity rate was much higher in *K*. *pneumoniae* (76%) than *E*. *coli* (45%). The overall gastrointestinal colonization rate of ESBL producing *Enterobacteriaceae* (ESBL-E) in hospitalized patients was 52% (95%CI; 46%–58%) of which, ESBL-E. *coli* and *K*.*pneumoniae* accounted for 68% and 32% respectively. Fecal ESBL-E carriage rate in neonates, children and adults was 74%, 59% and 46% respectively. Gastrointestinal colonization rate of ESBL-*E*.*coli* in neonates, children and adults was 11%, 42% and 42% respectively. Of all *E*. *coli* strains isolated from adults, children and neonates, 44%, 49% and 22% were ESBL positive (p = 0.28). The prevalence of ESBL-*K*.*pneumoniae* carriage in neonates, children and adults was 68%, 22% and 7% respectively. All *K*. *pneumoniae* isolated from neonates (100%) and 88% of *K*. *pneumoniae* isolated from children were ESBL positive, but only 50% of *K*.*pneumoniae* isolated from adults were ESBL positive (p = 0.001). Thirteen patients (5%) were carriers of both ESBL-*E*.*coli* and ESBL-KP. The overall carrier rate of ESBL producing isolates resistant to carbapenem was 2% (5/267), all detected in children; three with *E*.*coli* HL cephalosporinase (AmpC), resistant to ertapenem and two with K. *pneumoniae* Carbapenemase (KPC) resistant to meropenem, ertapenem and impenem. We report a high gastrointestinal colonization rate with ESBL-E and the emergence of carbapenems-resistant *K*. *pneumoniae* in Ethiopia. Urgent implementation of infection control measures, and surveillance are urgently needed to limit the spread within healthcare facilities and further to the community.

## Introduction

Antimicrobial resistance (AMR) in healthcare facilities is a global public health problem causing prolonged hospital stay, high cost of therapy and increased mortality [1]. Mobility of people, contaminated food and animals are main factors in the globalization of public health threats including spreading of resistant organisms [2]. Most importantly international travel, particularly to Africa, India and south East Asia appears to be a risk factor for colonization with ESBL-producing *E*. *coli* [3–5]. Although AMR is a global problem`, the impact is higher in Sub-Saharan Africa (SSA) due to limited available resources for healthcare infrastructure and wide irrational use of antimicrobial agents. Of all those who take antibiotics`, more than one-third do not get prescriptions from a doctor`, and about a quarter obtain antibiotics from an informal dispenser [6]. Although AMR is a global problem, the impact is higher in Sub-Saharan Africa (SSA) due to limited available resources for healthcare infrastructure and wide irrational use of antimicrobial agents. Of all those who take antibiotics, more than one-third do not get prescriptions from a doctor, and about a quarter obtain antibiotics from an informal dispenser [6]

ESBL-producing *Enterobacteriaceae* (ESBL-E), particularly *Escherichia coli* (ESBL-E.*coli*) and *Klebsiella pneumoniae* (ESBL-KP) are of major concerns due to concomitant multidrug resistance. The recent WHO report on AMR global surveillance indicate very high rates of resistant bacteria that cause common health-care associated and community-acquired infections worldwide [1]. The burden of ESBL-E and their respective antimicrobial resistance patterns are intensively studied in developed countries. However, data on the colonization rate with ESBL producing isolates, particularly on antibacterial resistance from developing countries particularly from sub Saharan Africa is yet limited mainly due to resource constrains [1, 7]. Available reports indicate that ESBL-producing *Enterobacteriaceae* in hospital and community settings in Africa is common, and the reported colonization rates as well as the resistance pattern vary between different countries with in the continent [8–12]. Considering the limited diagnostic and treatment possibilities in resource-poor countries, reliable surveillance data on antibacterial resistance in different African countries including Ethiopia is urgently required to raise awareness, map the burden and the resistance pattern for effective intervention measures, treatment recommendations and to identify emerging threats.

Transmission of antimicrobial resistant pathogens in healthcare facilities from former patients, visitors and healthcare providers may lead to outbreaks and spread to the community. In Ethiopia, initial studies reported high prevalence of hospital-acquired infections manly due to multi drug resistant pathogens at the countries’ largest tertiary referral Hospital [13, 14]. Majority of the isolated bacteria were gram-negative mostly resistant to the commonly used antimicrobials. Later a high level (63%) of hospital acquired multi-drug resistant bacterial infection was detected for many years [15]. Indeed, in the same hospital where we performed the current study and other hospitals in Ethiopia, infection outbreaks including neonatal sepsis due to multi-drug resistant bacteria, particularly in the neonatal units with a high mortality rate (41%) has been reported [16–18].

Surveillance and antimicrobial resistance data on ESBL-E is imperative, particularly in hospital environment to identify and prevent outbreaks. Furthermore the emergence and spread of carbapenem-resistant ESBL-E has become a growing threat globally and data from sub-Saharan Africa countries, including Ethiopia is lacking [1, 19, 20]. The objectives of this cross-sectional point prevalence study were, therefore, i) to investigate the gastrointestinal colonization rates and risk factors for ESBL producing *Enterobacteriaceae* in hospitalized patients ii) to identify the antimicrobial resistance profile of ESBL producing and non-ESBL producing E.coli and Klebsiella species iii) to perform phenotype characterization of ESBL producing *Enterobacteriaceae* strains particularly *E*. *coli* and *K*. *pneumoniae* in patients admitted at Ethiopia’s largest tertiary referral Hospital.

## Methods

### Study Design and Population

This cross-sectional descriptive point surveillance study was conducted during December 2012 at Tikur Anbessa Specialized Hospital (TASH), a 500-bed tertiary university teaching Hospital in Addis Ababa, Ethiopia. TASH is the biggest teaching University affiliated Hospital in Ethiopia, where patients from different parts of the country are refereed for further management. The Hospital provides service to both outpatients and inpatients admitted to medical, surgical, obstetric & gynecologic, orthopedic, neonatal and pediatric wards. All available in patient wards, including neonatal and ICUs as well as acute care wards and emergency clinics, where patients were admitted for ≥ 48 hours were eligible for study ward inclusion.

Inclusion criteria for study participants were; all hospitalized patients including neonates (newborn infants under 28 days of age), infants, children and adult patients who were admitted for ≥ 48 hours. Patients undergoing same day treatment or surgery during the survey date, patients seen at the outpatient department, patients in the emergency room monitored for < 48 hours, patients in long-term care wards and nursing homes were excluded.

The study protocol, including the consent procedure, received ethics approval from Institutional Review Boards (IRBs) of the College of Health Sciences, Addis Ababa University and Karolinska Institutet in Stockholm, Sweden. Written informed consent was obtained from both adult study participants as well as from parents or guardians on the behalf of the children and newborn infants who participated in the study. The study was conducted as per guidelines laid in the International Conference of Harmonization for Good Clinical Practice.

### ESBL Identification and Antimicrobial Susceptibility Testing

A single fecal sample/swab was collected from 267 hospitalized patients who were hospital admitted for ≥ 48 hours. All samples were cultured on CHROMagar™ ESBL (CHROMagar, Paris, France). Bacterial identification and antibiotic susceptibility testing were performed using the Vitek-2 system (bioMérieux, France). Susceptibility testing for 16 antimicrobials, namely ceftazidime (CAZ), cefotaxime (CTX), ciprofloxacin (CIP), cephalexin (CN), amoxicillin/clavulanic acid (AMC), amikacin (AN), aztreonam (ATM), ertapenem (ETP), cefoxitin (FOX), gentamicin (GM), imipenem (IPM), meropenem (MEM), tigecycline (TGC), tobramycin (TM), trimethoprim (TMP), piperacillin/tazobactam (TZP), was performed by using VITEK-2 AST card (AST-N218), which also has screening for ESBL. Interpretation of AST result was done according to EUCAST guidelines. *E*.*coli* ATCC 25922 and *K*. *pneumoniae* ATCC 700603 were used for quality control.

### Phenotype Characterization

Phenotypic characterization and categorization of ESBL positive *E*. *coli* and *K*. *pneumoniae*—was done using Neo-Sensitabs™ (Rosco, Denmark) according to the user′s guide. Briefly, Mueller Hinton agar plates were inoculated with a 0.5 McFarland adjusted suspension in 0.9% NaCl, streaked with cotton swabs using a plate rotator (bioMérieux S.A., Marcy l′Etoile, France), and incubated overnight at 35°C for 16–18 h. Differences in zones of inhibitions between a β-lactam alone compared with the combination with a β-lactamase inhibitor, as well as subjective synergy observations were determined for all ESBL positive isolates from Vitek-2 characterized *Enterobacteriaceae* strains.

All ESBL positive isolates resistant to CTX and CAZ were tested for ESBL_A_- using cefotaxime (CTX30), cefotaxime + clavulanic acid (CTX+C), ceftazidime (CAZ30) and ceftazidime + clavulanic acid (CAZ + CL). All ESBL positive isolates resistant to CTX or CAZ and FOX were tested for ESBL_M_–test, AmpC (Neo-Sensitabs confirm ID KIT ROSC98007) containing cefotaxime (CTX30), cefotaxime + cloxacillin (CTXCX), ceftazidime (CAZ30) and ceftazidime + cloxacillin (CAZCX).

The detection of putative carbapenemase production was first based on an initial phenotypic screen for carbapenem resistance, followed by the phenotypic characterization using ROSCO Neo-Sensitabs as a confirmatory test (Neo-Sensitabs confirm ID KIT ROSC98006), which consists of Meropenem (MRP10), Meropenem + Dipicolinic acid (MRPDP), Meropenem + Boronic acid (MRPBO) and Meropenem + cloxacillin (MRPCX). *K*. *pneumoniae* CCUG 56233, *K*. *pneumoniae* CCUG 56233 and *K*. *pneumoniae* CCUG 58547 were used as positive control strains for AmpC and porin loss, *K*. *pneumoniae* carbapenemase (KPC) and Metallo-β-lactamase (MBL) respectively. *K*. *pneumoniae* ATCC 25955 was used as negative control in all phenotype tests.

### Statistical Analyses

Multidrug resistance (MDR) in *Enterobacteriaceae* were defined as non-susceptible to ≥1 agent in >3 antimicrobial categories as listed by the international expert proposal for interim standard definitions [21]. Prevalence of ESBL producing isolates along with the corresponding 95% CI stratified by age group is presented by calculating frequencies and percentages. Chi-square test was used with appropriate correction for the observation. Pearson Correlations test and logistic regression analysis were used to identify risk factors for colonization by ESBL-E. Statistical analyses were performed using SPSS Statistics (IBM Corporation, Somers, NY) software, version 22.0. P values < 0.05 were considered to be statistically significant.

## Results

All adults, children and neonatal (newborn infants under 28 days of age) patients hospitalized at TASH (n = 398) during 10–20 December 2012 was approached to participate in this study. A total of 347 hospitalized patients fulfilled the study inclusion criteria, of which 267 patients (77%; 128 women and 139 men) who were willing to participate and able to provide fecal sample or rectal swab were enrolled. There were 154 adults (age span = 14 to 75 years old, median = 35 years, inter quartile range = 26 to 50 years), 94 children (age span = 1 month to 12 years old, median = 7 years, inter quartile range = 2 to 8 years old) and 19 neonates (age span 3 to 23 days; median = 9 days, inter quartile range = 4 to 13 days).

A total of 295 *E*.*coli and Klebsiella species* were isolated from 267 fecal samples. *E*.*coli* was the most common gram-negative organism identified (n = 235, 79.7%) followed by *K*.*pneumoniae* (n = 58, 19.7%) and K.*oxytoca* (n = 2, 0.7%). Of all ESBL-E isolates, 151 (51.2%) were ESBL positive, of which 70.2% (n = 106) were *E*.*coli*, 29.1% (n = 44) were *K*.*pneumoniae* and 0.7% (n = 1) were *K*. *oxytoca*.

Out of the total 235 E. *coli* strains isolated from fecal samples, 106 (45%) of them were ESBL-producing E. *coli* (95% CI = 39%–56%). Of all *E*. *coli* strains isolated from adults and children, 44% (65/147) and 49% (39/79) respectively were ESBL ESBL-producing E. *coli*. In contrast only 22% (2/9) of *E*. *coli* strains isolated from neonates were ESBL-producing E. *coli* (p = 0.28). On the other hand the distribution of ESBL-producing KP among the different age groups was quite different from what was observed for ESBL-producing E. coli. The overall prevalence of ESBL positivity among the 58 *K*. *pneumoniae* isolates was 75.9% (44/58), 95% CI = 63.6%-85.6%. All *K*. *pneumoniae* isolated from neonates (100%, 12/12) and 88% (21/24) of *K*. *pneumoniae* isolated from children were ESBL producing *K*.*pneumoniae*, but in adults only 50% (11/22) of *K*.*pneumoniae* were ESBL producing *K*.*pneumoniae* (p = 0.001).

**Fig 1:**
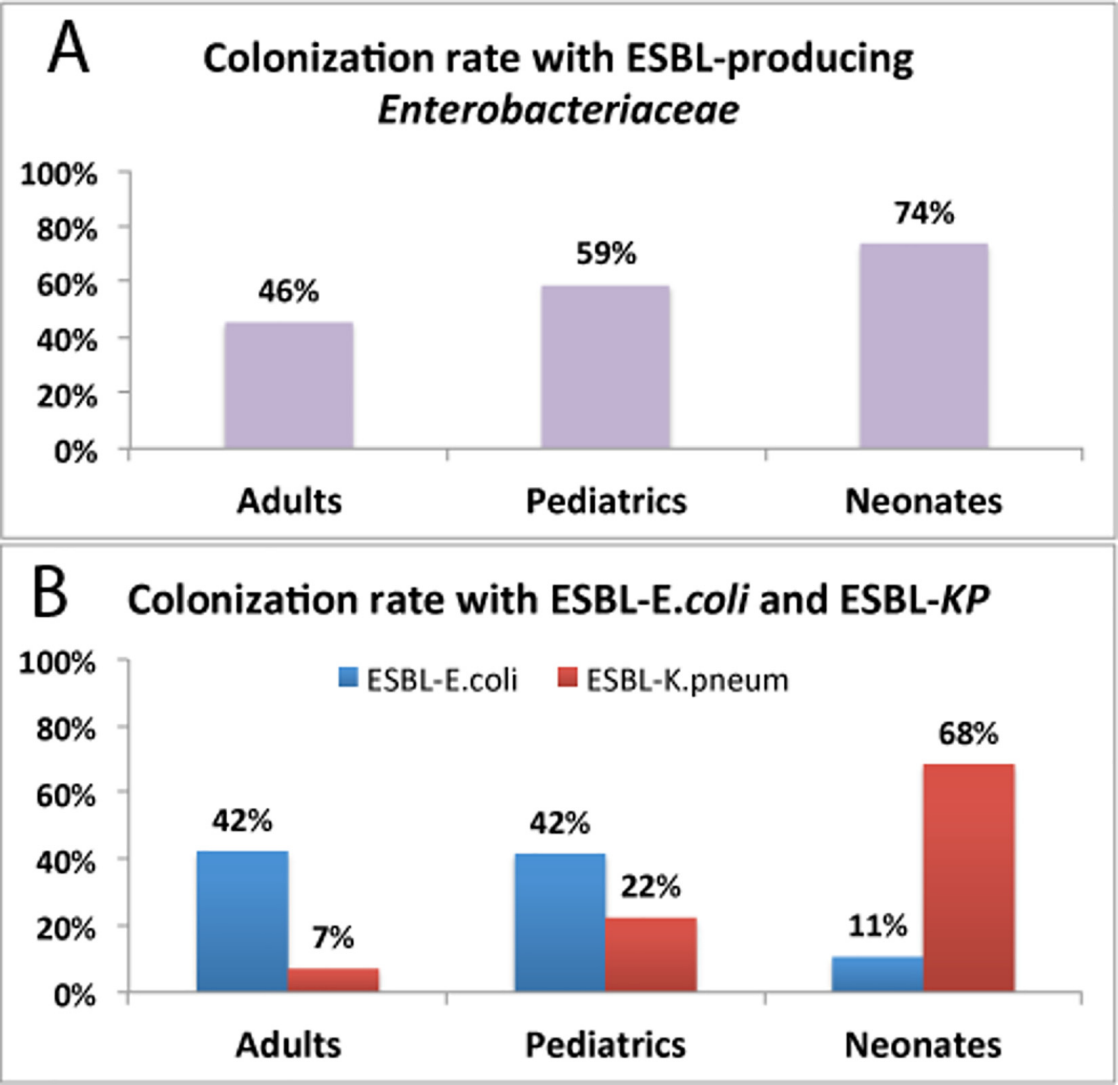
Colonization rate of ESBL producing *Enterobacteriaceae* (Top) and distribution of ESBL positive *E*. *coli* and *K*. *pneumonia* (bottom) in fecal samples collected from 267 hospitalized patients (154 adults, 94 children and 19 neonates). Thirteen out of 267 patients (5%) were carriers of both ESBL producing *K*.*pneumoniae* and *E*. *coli* (7 adults, 5 children and 1 neonates).

One hundred thirty-nine patients (52.1%; 95% CI = 46.1%–58.0%) out of 267 were carrier of ESBL producing *Enterobacteriaceae*. Comparison of rates of total ESBL producing isolates (positive for ESBL producing E.*coli* or *K*. *pneumoniae*) among adult, children and neonates is presented in Table 1 and Fig 1A. Out of the 139 patients, who were tested positive for ESBL-E, 95 of them (68.3%; 95% CI = 60.6%–76.0%) were colonized by ESBL-*E*. *coli*, 43 (31.0%; 95% CI = 23.3%–38.7%) by ESBL-*K*. *pneumoniae*, and 1 (0.4%) by ESBL-*K*. *oxytoca*. Prevalence of colonization with ESBL-E was significantly (p = 0.02) higher in neonates (74%) followed by children (59%) than in adults (46%). Logistic regression analysis indicted that having lower age as a significant risk factor for colonization by ESBL-E (p = 0.02). Neonates (p = 0.02, odds ratio = 3.360, 95% CI for odds ratio = 1.153 to 6.788) and children (p = 0.047, odds ratio = 1.692, 95% CI for odds ratio = 1.008 to 2.843) had a higher risk for being colonized by ESBL-E compared to adult patients.

**Table 1:** Gastrointestinal colonization rate of ESBL positive (ESBL+ve) and negative (ESBL-ve) isolates in hospitalized patients and stratified by patient age group, at Tikur Anbessa Specialized hospital, December 2012.

Organism	ESBL positivity	All (n = 267)	Patient age group			p value
			Adult (n = 154)	Children (n = 94)	Neonates (n = 19)	
*Total*	ESBL -ve	128 (47.9%)	84 (54.5%)	39 (41.5%)	5 (26.3%)	0.02
	ESBL +ve	139 (52.1%)	70 (45.5%)	55 (58.5%)	14 (73.7%)	
*E*. *coli*	ESBL -ve	161 (60.3%)	89 (57.8%)	55 (58.5%)	17 (89.5%)	0.026
	ESBL +ve	106 (39.7%)	65 (42.2%)	39 (41.5%)	2 (10.5%)	
*K*. *pneumoniae*	ESBL -ve	222 (83.1%)	143 (92.9.0%)	73 (77.7%)	6 (31.6%)	< 0.0001
	ESBL +ve	45 (16.9%)	11 (7.1%)	21 (22.3%)	13 (68.4%)	

Fecal colonization rate of ESBL producing *Enterobacteriaceae* in hospitalized patients stratified by ward specialty is presented in Table 2. Gastrointestinal colonization rates by ESBL-E were much higher for patients admitted at intensive care unit, medical and emergency wards. There was a significant positive correlations of high colonization rates by ESBL-E with higher maximum bed capacity per room and increasing number of patients admitted in single room and (Pearson Correlations test p < 0.0001). Having more than two patients in a single room was a significant risk factor for being colonized by ESBL-E (p < 0.0001, odds ratio = 4.017, 95% CI for odds ratio = 2.255 to 5.348).

**Table 2:** Fecal colonization rate of ESBL producing *Enterobacteriaceae* in hospitalized patients stratified by ward specialty during December 2012 at Tikur Anbessa Specialized Hospital in Addis Ababa, Ethiopia.

Patents age group	Ward specialty	ESBL producing Enterobacteriaceae			
		Negative		Positive	
		N	%	N	%
Adult	Emergency	0	0.0%	2	100.0%
	Gynecology & obstetrics	27	79.4%	7	20.6%
	Medical general	16	30.2%	37	69.8%
	Medical ICU	0	0.0%	1	100.0%
	Orthopedics	11	42.3%	15	57.7%
	Oncology	12	85.7%	2	14.3%
	Surgery	18	75.0%	6	25.0%
Neonates	Neonates	5	26.3%	14	73.7%
Pediatrics	Emergency	7	31.8%	15	68.2%
	ICU	0	0.0%	2	100.0%
	Medical general	10	23.8%	32	76.2
	Surgical	22	78.6%	6	21.4%

**Fig 2:**
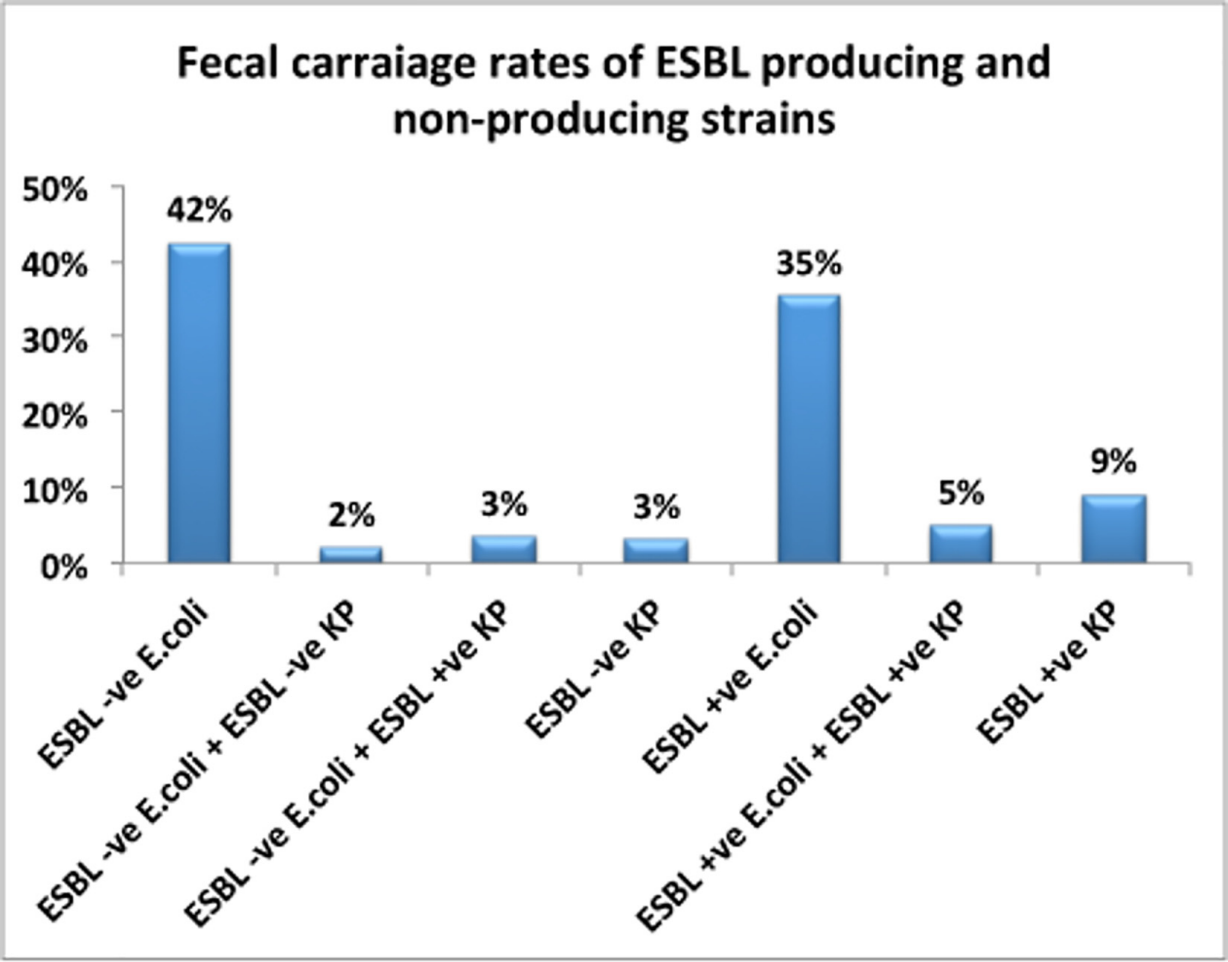
Distribution of gastrointestinal colonization rates of *E*. *coli* and *K*.*pneumoniae* stratified by presence (+ve) and or absence (-ve) of ESBL producing isolates in all hospitalized patients (n = 267).

The overall prevalence of colonization by ESBL positive *E*.*coli* (ESBL-E.*coli*) among 267 hospitalized patients was 40% (95% CI = 34.1%–45.9% whereas 45 patients (17%; 95% CI = 12.5%–21.5%) were carriers of ESBL-*K*.*pneumoniae* (p < 0.0001). There was a significant difference between age groups and type of ESBL producing strains (Fig 1B). Colonization with ESBL-*E*.*coli* was more common in adults (42%) and children (42%) than neonates (11%). In contrast, colonization with ESBL positive *K*. *pneumoniae* (ESBL-KP) was much higher (p < 0.0001) in neonates (68%) than in children (22%) or adults (7%). All Klebsiella species isolated from neonates were ESBL positive. Where as 64% and 50% of all Klebsiella species isolated from children and adults were ESBL positive respectively. Distribution of gastrointestinal *E*. *coli* and *K*.*pneumoniae* carriage rates stratified by presence and or absence of ESBL producing isolates in all hospitalized patients is presented in Fig 2. Twenty-eight patients (10%) were carriers of both *E*. *coli* and *K*. *pneumoniae*, and 13 patients (5%) were carriers of both ESBL positive *K*. *pneumoniae* and ESBL-*E*.*coli* (seven adults, five children and one neonate).

### Antimicrobial Susceptibility of ESBL-E.*coli* and *K*.*pneumoniae*

**Fig 3:**
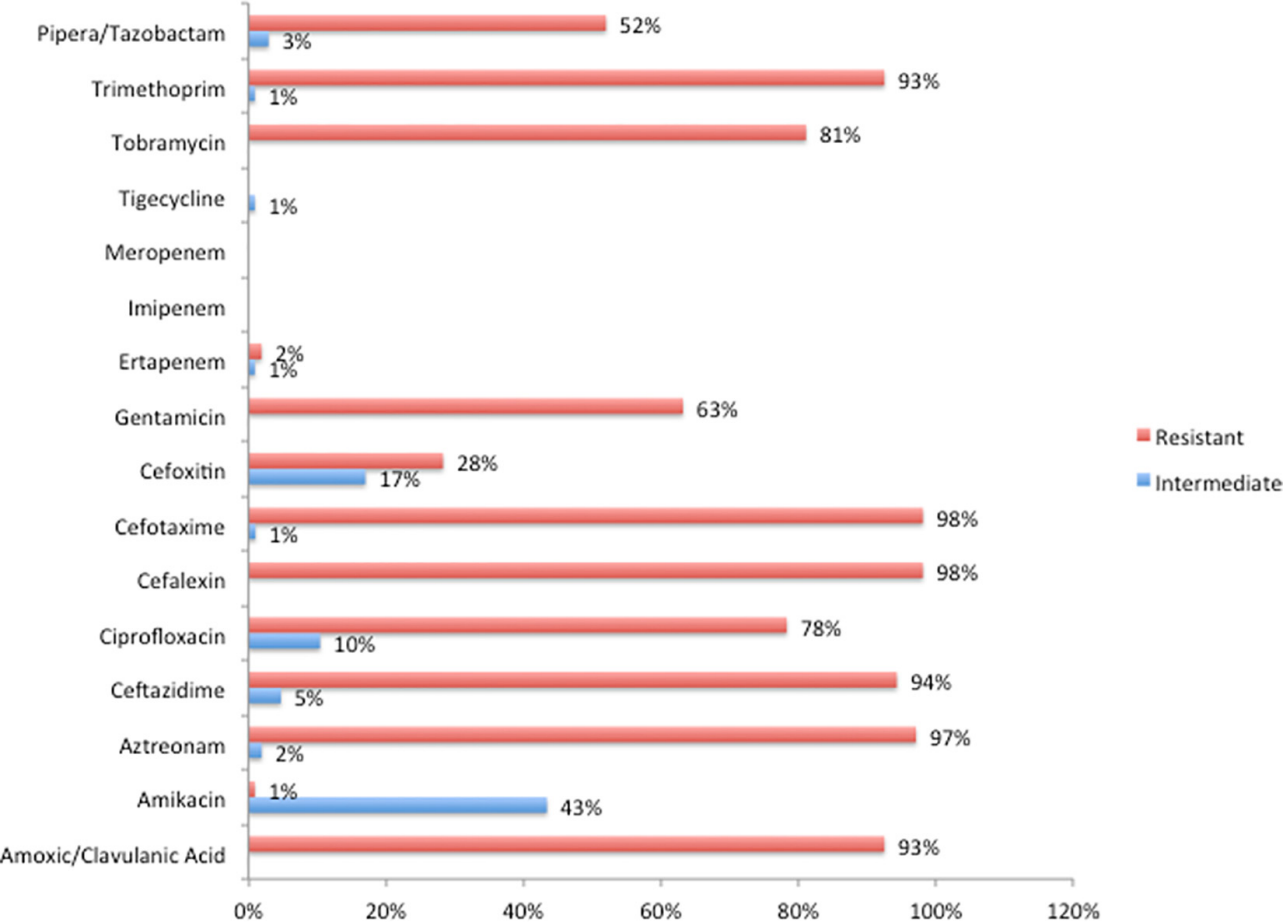
Antimicrobial resistance pattern of ESBL producing (n = 106) *E*.*coli* strains isolated from hospitalized patients fecal samples/swab. The over all prevalence of ESBL positivity among the 235 *E*.*coli* isolates was 45.1% (106/235).

**Fig 4:**
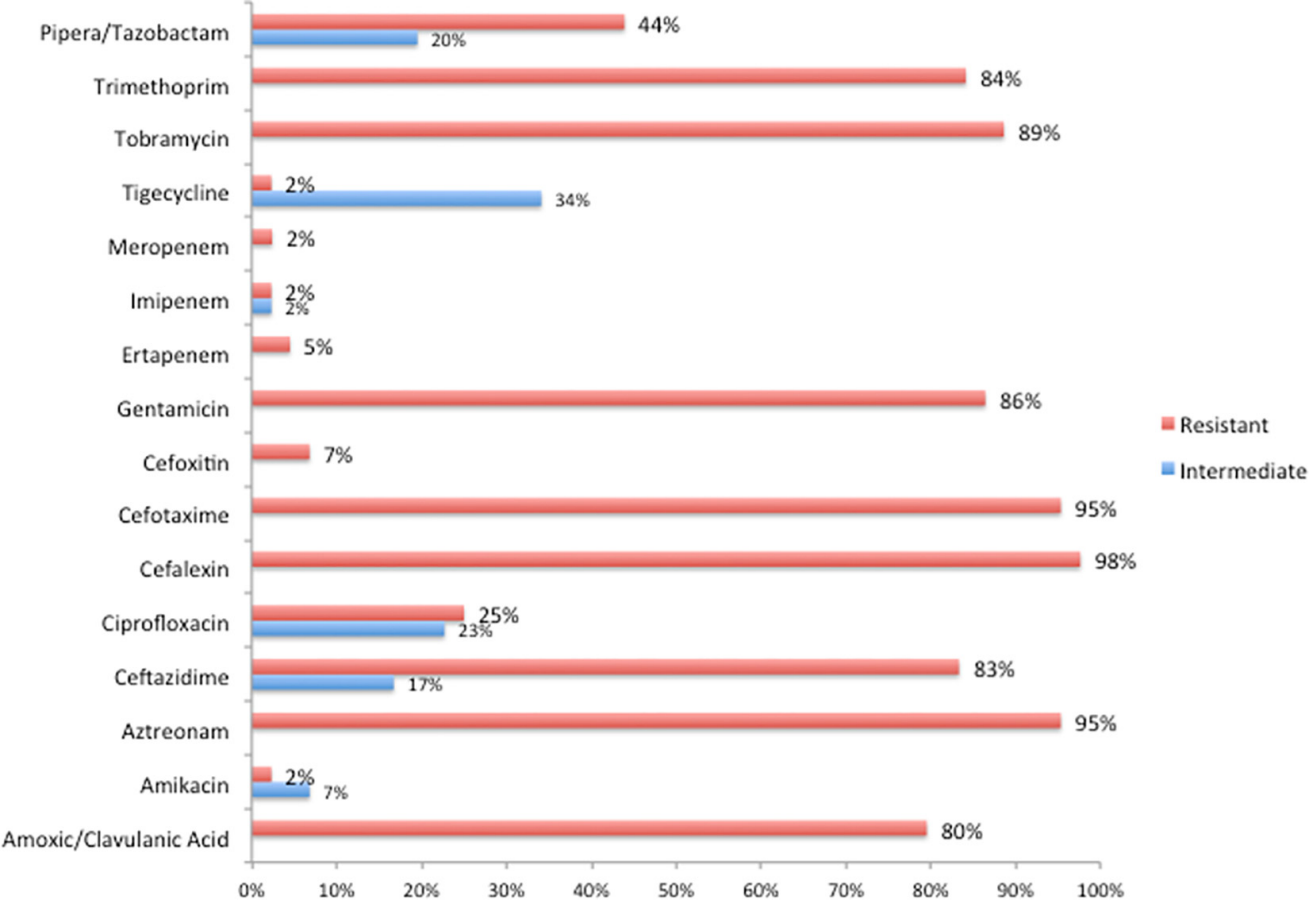
Antimicrobial resistance pattern of ESBL positive (n = 44) *K*.*pneumoniae* strains isolated from hospitalized patients fecal samples/swab. The prevalence of ESBL positivity among the 58 K.*pneumoniae* isolates was 76% (44/58).

Fig 3 shows the antimicrobial resistance pattern for ESBL positive (n = 106) *E*.*coli* isolates. All ESBL-*E*.*coli* were 100% sensitive to meropenem and imipenem, while 3% were resistant or intermediate resistant to ertapenem. Non-ESBL producing *E*. *coli* strains were more often resistant to amoxicillin/clavulanic acid, trimethoprim, and piperacillin/tazobactam. The highest resistance in ESBL negative isolates was seen for TMP (38%), amoxicillin/clavulanic acid (17%), and 12% were resistant to both drugs. All ESBL-*E*. *coli* were MDR but only 2% (4 out of the 175) of the non-ESBL-*E*.*coli* (n = 129; p < 0.0001). Fig 4 shows the comparison of antimicrobial resistance pattern for ESBL positive *K*. *pneumoniae*. All non-ESBL producing *K*. *pneumoniae* isolates were sensitive to all antimicrobial tested for, while all ESBL-KP were MDR (p < 0.0001).

### Prevalence of ESBL Producing and Carbapenem Resistant *Enterobacteriaceae*

Five children (1.9%, 5/267) were colonized with ESBL positive and carbapenem resistant Enterobacteriaceae (ESBL-CARBA)_._ Two children were colonized by ESBL and carbapenemase producing K. *pneumoniae* (KPC) with MBL+KPC: One child was colonized by ESBL producing *K*. *pneumoniae* isolate resistant to all antibiotics including ertapenem, imipenem, meropenem and the other child was colonized with *K*. *pneumoniae* resistant to both ertapenem and imipenem but sensitive to meropenem. The remaining three children were colonized by ESBL and carbapenemase producing *E*.*coli* with HL cephalosporinase (AmpC) phenotype. All ESBL-CARBA *E*. *coli* isolates were resistant to ertapenem but sensitive to imipenem and meropenem.

## Discussion

In the present study, we investigated gastrointestinal carriage rate and antimicrobial resistance pattern of ESBL producing *Enterobacteriaceae* in hospitalized patients admitted to Ethiopia’s largest tertiary hospital. We found high rates of colonization with ESBL-E (52%), of which, ESBL-*E*.*coli* accounted for 68% and the rest by ESBL-KP (31%). Although E. *coli* was the most frequently isolated than K. pneumoniae, ESBL production was more prevalent in K. *pneumoniae*. Among *E*. *coli* isolates, 40% were ESBL positive, while the corresponding figure for *K*. *pneumoniae* was 76%. Our result indicates having lower age and higher number of beds in a single room as a risk factor for being colonized by ESBL-E.

Fecal carriage of ESBL producing organisms in hospitalized patients has shown large variations worldwide [8–12, 22, 23]. The overall fecal carriage rates of ESBL-producing *Enterobacteriaceae* in our study from Ethiopia’s largest referral hospital (52%) is comparable to the overall pooled ESBL proportion estimate for East African hospitals (42%) [11], Cameron (55.3%)[24] and Ghana (49.3%) [25], but it is much higher than reports from Europe (5.4–25%) and the United States (1–25%) [22, 23]. However the colonization rates of ESBL-producing *Enterobacteriaceae* among hospitalized neonates (74%) and children (59%) in this study is much higher than reports from other paediatric or neonatal clinics from elsewhere, including other African countries [7] such as Gabon (45% in children) [26], Ghana (65.1% in neonates) [25], Guinea-Bissau (32.6% in children)[27] and Ecuador (56% in neonates) [28]. Recently a high rate of fecal carriage of ESBL-E (59%) in healthy children from Bangui, Central African Republic is reported [10]. Previously based on a systematic review of old data, Tansarli et.al, reported that proportion of ESBL-producing *Enterobacteriaceae* may not be high in Africa [7]. However the available recent data from different African courtiers including ours indicates a much higher burden ESBL-E colonization rate in African countries than previously reported by Tansarli et al, [7].

ESBL-producing *Enterobacteriaceae* infections is a growing threats to infants and children [29]. Treatment of neonatal sepsis has become a challenge with the emergence of carbapenemase-producing bacteria [30]. In our study, *K*. *pneumoniae* isolated from neonates and children less than five years of age had a significantly higher proportion of ESBL positivity than those isolated from adults. We found a significant association between a lower age and high colonization rate with ESBL producing *K*. *pneumoniae*. Therefore longer stay at neonatal or pediatric unit could be risk factor for colonization with ESBL-KP at TASH. Hospital-borne babies in developing countries are at high risk of neonatal infections because of poor compliance to intrapartum and postnatal infection-control practices [31]. Previous reports indicate that ESBL producing *E*. *coli* colonized mothers colonized with ESBL producing *E*. *coli* are an independent risk factor and potential reservoir for transmission of ESBL-E to neonates [9, 32]. Outbreaks caused by ESBL-producing bacteria such as *K*. *pneumoniae* in neonatal intensive care units are well described [33]. Indeed, in the same hospital where we performed the current study, infection outbreaks including neonatal sepsis due to multi-drug resistant bacteria, particularly in the neonatal units with a high neonatal mortality (41%) has been reported [16–18]. ESBL-E colonized children and other patients with underlying conditions could also act as vectors of microorganisms between the hospital and the community [34].

The antimicrobial resistance profile between the different isolates (Figs 3 and 4) is quite comparable and this may suggests local transmission of resistant strains with in the hospital. High levels of resistances for aztreonam, amoxicillin/clavulanic acid, aztreonam, cefotaxime, cefalexin, ceftazidime, trimethoprim and tobramycin were observed for both ESBL producing E. *coli* and K. *pneumoniae*. Poor hand hygiene practice, infection control measures, multi-bed rooms and crowded patients in a single rooms that were noticed during the study period might have contributed for cross-transmission of resistant strains with in the Hospital. Treatment of severe infections caused by ESBL producing *E*. *coli* and *K*. *pneumoniae* in hospitalized patients rely on carbapenems, which are the last resort to treat life-threatening infections. Infections with carbapenemase-producing *Enterobacteriaceae* are most difficult to manage and associated with high mortality rates [35]. The recent WHO report on AMR global surveillance indicates the identification of K. *pneumoniae* resistant to carbapenems in most of the countries that provided data, with reported proportions of resistance reaching up to 54% [1]. To the best of our knowledge this is the first report to indicate the emergence of colonization with carbapenems resistant ESBL producing strains in Ethiopia. Our finding of ESBL producing *K*. *pneumoniae* isolates resistant to all other antibiotics effective for treatment including carbapenems is worrisome. These extensively resistant isolates were susceptible only to tigecycline, a newer glycylcycline, recommended as option for the management of infections with carbapenems-resistant *Enterobacteriaceae*. However, tigecycline is currently not available in public Hospitals in Ethiopia, which poses a great challenge for patient management. Currently the burden of carbapenem resistant ESBL strains is not known and needs to be further investigated.

The emergence and spread of AMR pathogens is a threat for many African countries including Ethiopia. ESBL colonization may predispose the patient to recurrent infections and cross-transmission to others [9, 32, 36, 37]. Patients colonized with ESBL are at increased risk for invasive infections compared with non-colonized patients [36, 38]. The two children colonized with carbapenem-resistant ESBL-KP were admitted with other sick children in the same room with close proximity. If hand hygiene is not applied as recommended, these resistant bacteria can spread from one patient to another via healthcare workers contaminated hands. In hospitals, identification of patients colonized or infected with ESBL and adoption of subsequent preventive measures (isolation in a single-patient room) is suggested to prevent cross-transmission and reduce morbidity and healthcare costs.[39, 40] However, screening and contact isolation of patients colonized with ESBL in developing countries is usually not feasible due to resource constrain. Thus, effective intervention measures such as hand hygiene to prevent cross-contamination and rational use of antimicrobial are the priority options in Ethiopia.

Antimicrobial susceptibility testing (AST) is the best method to guide antimicrobial prescription. But it is not available routinely outside of tertiary referral hospitals in most African countries including Ethiopia. Furthermore, patients have to pay out of their pocket for the analysis, which further hampers its implementation in clinical management. As a result data on antimicrobial susceptibility pattern in hospital and community acquired infections are very scarce, despite their potential to reduce neonatal and children mortality [41]. Even in tertiary hospitals, the current practice of identifying infection is by clinical symptoms and in most cases the medical doctors prescribe antibiotics without prior culture and AST results due to lack of resources. Our result provides valuable data on the resistance pattern of ESBL producing *E*. *coli* and *K*. *pneumoniae* in Ethiopia.

The prevalence of infections caused by ESBL- producing bacteria is increasing worldwide. Thus Global AMR surveillance is needed, since population mobility is the main factor for its spread [2, 42, 43]. A global surveillance system for antimicrobial resistance is developed by WHO in support of the global action plan against antimicrobial resistance accepted at the World Health Assembly 2015 [44]. To monitor the effect of interventions, AMR surveillance should be integrated in national plans that should be developed to combat AMR, as stipulated in the WHO global action plan [44].

Limitation of the study includes lack of clinical data as a risk factor and molecular characterization of the resistant strains. The primary objective and design of this study was to perform initial baseline surveillance to identify the burden and resistant pattern of ESBL producing *Enterobacteriaceae* in hospitalized patients. Result from this study provides valuable baseline information to conduct further large prospective study involving molecular characterization of resistant strains to identify the type, source and spread pattern of antimicrobial resistance in the Hospital.

In conclusion, we report high rates of intestinal colonization rate with ESBL producing multi-drug resistant *Enterobacteriaceae* and the emergence of ESBL-producing carbapenems resistant K. *pneumoniae* particularly among pediatric patients in Ethiopia. Routine infection preventions strategies such as compliance to hand hygiene principles, rational use of antimicrobial agents and surveillance of AMR are urgently needed to prevent and control the spread of antimicrobial-resistant pathogens in the healthcare facilities.
